# Supplementary material for: Quantitative and qualitative evaluation of the impact of the G2 enhancer, bead sizes and lysing tubes on the bacterial community composition during DNA extraction from recalcitrant soil core samples based on community sequencing and qPCR
Source: PLoS One. 2019 Apr 11;14(4):e0200979. doi: 10.1371/journal.pone.0200979 (PMC6459482; doi:10.1371/journal.pone.0200979)
Supplement: S1 Table — (PDF) [file pone.0200979.s001.pdf]

S1 Table. Qubit measurements (ng/µl).

**Serie n**	Serie 1	Serie 2	Serie 3	Serie 4	Serie 5	Serie 6	Serie 7	Serie 8	Serie 9	Negative control	
**Tube type**	Fast tube	Fast tube	Fast tube	Fast tube	Ampliqon	Ampliqon	Ampliqon	Ampliqon	Ampliqon	Fast tube	Fast tube
**Beads size**	Mixed	Mixed	1.4 mm	0.1 mm	1.4 mm	0.1 mm	1.4 mm	0.1 mm	Mixed	Mixed	Mixed
**With/Without G2**	Without G2	With G2	Without G2	Without G2	With G2	With G2	Without G2	Without G2	Without G2	With G2	Without G2
**1a**	0.737	1.180	0.373	0.272	1.140	0.554	0.261	0.222	0.478	-	-
**2a**	0.567	1.090	0.324	0.314	1.020	0.517	0.338	0.323	0.556	-	-
**3a**	0.543	1.300	0.356	0.263	1.010	0.535	0.315	0.288	0.507	-	-
**4a**	0.473	1.180	0.399	0.319	0.935	0.565	0.350	0.240	0.518	-	-
**5a**	0.639	1.110	0.346	0.301	0.987	0.423	0.306	0.261	0.457	-	-
										-	-
**1b**	0.700	1.400	0.355	0.273	1.030	0.606	0.318	0.235	0.528	-	-
**2b**	0.735	1.310	0.320	0.353	1.810	0.472	0.363	0.336	0.545	-	-
**3b**	0.694	1.350	0.308	0.267	1.040	0.583	0.268	0.274	0.457	-	-
**4b**	0.630	1.300	0.363	0.259	0.929	0.581	0.366	0.222	0.621	-	-
**5b**	0.669	1.170	0.328	0.279	1.030	0.537	0.289	0.254	0.469	-	-
										-	-
**1c**	0.807	1.520	0.363	0.242	1.190	0.771	0.294	0.173	0.542	-	-
**2c**	0.865	1.250	0.356	0.368	1.210	0.569	0.418	0.361	0.567	-	-
**3c**	0.688	1.590	0.289	0.307	1.020	0.585	0.269	0.268	0.456	-	-
**4c**	0.682	1.300	0.390	0.290	1.100	0.672	0.337	0.212	0.454	-	-
**5c**	0.774	1.130	0.269	0.311	0.975	0.597	0.266	0.242	0.334	-	-
**Average 1a:5a**	0.5918	1.172	0.3596	0.2938	1.0184	0.5188	0.314	0.2668	0.5032	-	-
**Average 1b:5b**	0.6856	1.306	0.3348	0.2862	1.1678	0.5558	0.3208	0.2642	0.524	-	-
**Average 1c:5c**	0.7632	1.358	0.3334	0.3036	1.099	0.6388	0.3168	0.2512	0.4706	-	-
**Average 1a;1b;1c**	0.748	1.367	0.364	0.262	1.120	0.644	0.291	0.210	0.516	0.050	<D.L.
**Average 2a;2b;2c**	0.722	1.217	0.333	0.345	1.347	0.519	0.373	0.340	0.556	0.115	0.060
**Average 3a;3b;3c**	0.642	1.413	0.318	0.279	1.023	0.568	0.284	0.277	0.473	0.086	<D.L.
**Average 4a;4b;4c**	0.595	1.260	0.384	0.289	0.988	0.606	0.351	0.225	0.531	0.104	0.080
**Average 5a;5b;5c**	0.694	1.137	0.314	0.297	0.997	0.519	0.287	0.252	0.420	0.121	
**Average**	0.68	1.28	0.34	0.29	1.10	0.57	0.32	0.26	0.50	0.095	0.07
**STD**	0.0153	0.0502	0.0037	0.0039	0.0901	0.0119	0.0070	0.0105	0.0115	0.0033	0.0002

Series 1 to 9 corresponding to five individual samples, measured in triplicates (a, b and c)
