# Supplementary material for: Quantitative and qualitative evaluation of the impact of the G2 enhancer, bead sizes and lysing tubes on the bacterial community composition during DNA extraction from recalcitrant soil core samples based on community sequencing and qPCR
Source: PLoS One. 2019 Apr 11;14(4):e0200979. doi: 10.1371/journal.pone.0200979 (PMC6459482; doi:10.1371/journal.pone.0200979)
Supplement: S2 Table — (PDF) [file pone.0200979.s002.pdf]

S2 Table. Scheffe's score for Qubit dataset based on the difference series comparisons

Scheffe's Critic	First Comparison	Second Comparison	Scheffe's Score
**27.61***	FAST tube + Mix beads FAST + G2	FAST tube + Mix beads FAST - G2	**172.86**
	FAST tube + 1.4 mm beads - G2	FAST tube + 0.1 mm beads - G2	1.12
	FAST tube + Mix beads FAST - G2	FAST tube + 1.4 mm beads - G2	**55.01**
	FAST tube + Mix beads FAST - G2	FAST tube + 0.1 mm beads - G2	**71.78**
	Ampliqon tube + 1.4 mm beads + G2	Ampliqon tube + 1.4 mm beads - G2	**292.02**
	Ampliqon tube + 0.1 mm beads + G2	Ampliqon tube + 0.1 mm beads - G2	**46.50**
	Ampliqon tube + 1.4 mm beads + G2	Ampliqon tube + 0.1 mm beads + G2	**132.48**
	FAST tube + 1.4 mm beads - G2	FAST tube + 0.1 mm beads - G2	1.12
	Ampliqon tube + 1.4 mm beads - G2	Ampliqon tube + 0.1 mm beads - G2	1.54
	Ampliqon tube + Mix beads FAST - G2	FAST tube + Mix beads FAST - G2	15.80
	Ampliqon tube + 1.4 mm beads - G2	FAST tube + 1.4 mm beads - G2	0.31
	Ampliqon tube + 0.1 mm beads - G2	FAST tube + 0.1 mm beads - G2	0.55
	FAST tube + Mix beads FAST - G2	Ampliqon tube + Mix beads FAST - G2	15.80
	Fast Tube + Mixed Beads + Neg + G2	FAST tube + Mix beads FAST + G2	**675.95**

(*) Scheffe's Critic - calculated based on DNA amount
(172.86) Statistically significant
