# Supplementary material for: Quantitative and qualitative evaluation of the impact of the G2 enhancer, bead sizes and lysing tubes on the bacterial community composition during DNA extraction from recalcitrant soil core samples based on community sequencing and qPCR
Source: PLoS One. 2019 Apr 11;14(4):e0200979. doi: 10.1371/journal.pone.0200979 (PMC6459482; doi:10.1371/journal.pone.0200979)
Supplement: S3 Table — (PDF) [file pone.0200979.s003.pdf]

S3 Table. qPCR copy-number results (quantification based on 16s Gene/µl)

FAST tube + Mix beads FAST - G2	FAST tube + Mix beads FAST + G2	FAST tube + 1.4 mm beads - G2	FAST tube + 0.1 mm beads - G2	Ampliqon tube + 1.4 mm beads + G2	Ampliqon tube + 0.1 mm beads + G2	Ampliqon tube + 1.4 mm beads - G2	Ampliqon tube + 0.1 mm beads - G2	Ampliqon tube + Mix beads FAST - G2
239.674	533.510	150.081	136.344	344.158	197.511	146.488	122.612	306.625
288.782	474.536	146.095	119.145	361.691	217.290	147.103	99.747	278.049
278.633	373.218	156.656	86.502	447.643	139.696	135.779	104.346	257.049
357.835	441.625	120.722	168.695	460.500	174.806	168.359	161.059	289.533
310.236	711.104	161.245	126.655	417.669	188.429	134.722	141.191	286.099
324.357	541.500	98.555	114.850	436.516	190.285	142.007	143.750	271.976
265.700	547.510	154.716	126.569	383.342	246.825	139.759	128.784	266.401
271.138	608.738	158.943	78.312	347.810	193.563	143.349	128.784	275.977
264.020	545.876	169.024	89.805	266.555	211.566	157.162	128.784	278.869
Average								
288.931	530.846	146.226	116.320	385.098	195.552	146.081	128.784	278.953
Std.								
34.101	91.386	21.150	26.673	58.663	27.878	10.108	17.944	13.435
