# Supplementary material for: Quantitative and qualitative evaluation of the impact of the G2 enhancer, bead sizes and lysing tubes on the bacterial community composition during DNA extraction from recalcitrant soil core samples based on community sequencing and qPCR
Source: PLoS One. 2019 Apr 11;14(4):e0200979. doi: 10.1371/journal.pone.0200979 (PMC6459482; doi:10.1371/journal.pone.0200979)
Supplement: S4 Table — (PDF) [file pone.0200979.s004.pdf]

S4 Table. ANOVA table based on qPCR dataset

Source of Variation	SS	df	MS	F	P-value	F crit
Between Groups	$1.58179E^{+12}$	9	$1.75755E^{+11}$	93.2553326	$1.0147E^{-36}$	2.0090249
Within Groups	$1.39465E^{+11}$	74	1.884.663.581			
Total	$1.72126E^{+12}$	83				
