# Supplementary material for: Quantitative and qualitative evaluation of the impact of the G2 enhancer, bead sizes and lysing tubes on the bacterial community composition during DNA extraction from recalcitrant soil core samples based on community sequencing and qPCR
Source: PLoS One. 2019 Apr 11;14(4):e0200979. doi: 10.1371/journal.pone.0200979 (PMC6459482; doi:10.1371/journal.pone.0200979)
Supplement: S5 Table — (PDF) [file pone.0200979.s005.pdf]

S5 Table. Scheffe's score for qPCR dataset based on the difference series comparisons

Scheffe's Critic	First Comparison	Second Comparison	Scheffe's Score
**26.117**	Fast tube + Mix beads + G2	Fast tube + Mix beads	**139.74 a***
	Fast tube + 1.4 beads	Fast tube + 0.1 mm beads	2.14
	Fast tube + Mix beads	Fast tube + 1.4 mm beads	**48.62 b***
	Fast tube + Mix beads	Fast tube + 0.1 mm beads	**71.14 b***
	Ampliqon tube + 1.4 mm beads + G2	Ampliqon tube + 1.4 mm beads	**136.41 a***
	Ampliqon tube + 0.1 mm beads + G2	Ampliqon tube + 0.1 mm beads	10.64 a
	Ampliqon tube + 1.4 mm beads + G2	Ampliqon tube + 0.1 mm beads + G2	**85.78 d***
	Ampliqon tube + 1.4 mm beads	Ampliqon tube + 0.1 mm beads	0.71 d
	Ampliqon tube + Mix beads	Fast tube + Mix beads	0.24 e
	Ampliqon tube + 1.4 mm beads	Fast tube + 1.4 mm beads	0.00 e
	Ampliqon tube + 0.1 mm beads	Fast tube + 0.1 mm beads	0.37 e
	Fast tube + Mix beads	Ampliqon tube + Mix beads	0.24
	Fast Tube + Mix Beads + Neg + G2	Fast Tube + Mix Beads + G2	**334.82 c***
	Fast Tube + Mix Beads + Neg + G2	Fast Tube + Mix Beads + Neg	5.04 c

A result is considered to be statistically relevant when the Scheffe's score is higher than the Scheffe's critical value (Scheffe's Critic = 26.117). * Identify statistically relevant comparison, while letters identify the category in which they belong. **a**: Comparison based on presence/absence of G2. **b**: Comparison between mixed-beads and respectively 1.4 mm and 0.1 mm. **c**: Comparison between the series A-NEG+G2 and B. A-NEG+G2 and A-NEG (all reported in Table 1) to show that, based on the controls, G2 does not have any effect statistically relevant in terms of yield of DNA. **d**: Comparison between different bead-size (1.4 and 0.1 mm) in presence/absence of G2 **e**: Comparison between the different plastic tubes to evaluate the plastic-effect. **(139.74)** Statistically significant
