# Supplementary material for: Quantitative and qualitative evaluation of the impact of the G2 enhancer, bead sizes and lysing tubes on the bacterial community composition during DNA extraction from recalcitrant soil core samples based on community sequencing and qPCR
Source: PLoS One. 2019 Apr 11;14(4):e0200979. doi: 10.1371/journal.pone.0200979 (PMC6459482; doi:10.1371/journal.pone.0200979)
Supplement: S6 Table — (PDF) [file pone.0200979.s006.pdf]

S6 Table. Scheffe's test results as presented in S5 Table and used to produce Fig 3

G2	Beads	Tubes
139.74	2.10	0.00005
136.40	48.20	0.37
10.60	71.10	0.23
	**Averages**	
95.58	40.47	0.20

Data was used for Figure 3 preparation
