# Supplementary material for: Quantitative and qualitative evaluation of the impact of the G2 enhancer, bead sizes and lysing tubes on the bacterial community composition during DNA extraction from recalcitrant soil core samples based on community sequencing and qPCR
Source: PLoS One. 2019 Apr 11;14(4):e0200979. doi: 10.1371/journal.pone.0200979 (PMC6459482; doi:10.1371/journal.pone.0200979)
Supplement: S7 Table — (PDF) [file pone.0200979.s007.pdf]

S7 Table. ANCOM summary based on G2 comparison.

,Reject null hypothesis,W

Unassigned	__	__	__	__,False,0
k__Archaea	__	__	__	__,False,0
k__Archaea	p__Crenarchaeota	c__MBGA	__	__,False,0
k__Archaea	p__Crenarchaeota	c__MBGA	o__	f__,False,0
k__Archaea	p__Crenarchaeota	c__Thaumarchaeota	o__AK31	f__,False,0
k__Archaea	p__Crenarchaeota	c__Thaumarchaeota	o__Cenarchaeales	__,False,0
k__Archaea	p__Crenarchaeota	c__Thaumarchaeota	o__Cenarchaeales	f__,False,0
k__Archaea	p__Crenarchaeota	c__Thaumarchaeota	o__Cenarchaeales	f__Cenarchaeaceae,False,0
k__Archaea	p__Crenarchaeota	c__Thaumarchaeota	o__Nitrososphaerales	f__Nitrososphaeraceae,False,0
k__Archaea	p__Euryarchaeota	__	__	__,False,0
k__Archaea	p__Euryarchaeota	c__Thermoplasmata	o__E2	__,False,0
k__Archaea	p__Euryarchaeota	c__Thermoplasmata	o__E2	f__TMEG,False,0
k__Archaea	p__Euryarchaeota	c__Thermoplasmata	o__E2	f__[Methanomassiliicoccaceae],False,0
k__Archaea	p__[Parvarchaeota]	c__[Parvarchaea]	o__WCHD3-30	f__,False,0
k__Archaea	p__[Parvarchaeota]	c__[Parvarchaea]	o__YLA114	f__,False,0
k__Bacteria	__	__	__	__,False,0
k__Bacteria	p__Acidobacteria	__	__	__,False,0
k__Bacteria	p__Acidobacteria	c__	o__	f__,False,0
k__Bacteria	p__Acidobacteria	c__Acidobacteria-5	o__	f__,False,0
k__Bacteria	p__Acidobacteria	c__Acidobacteria-6	__	__,False,0
k__Bacteria	p__Acidobacteria	c__Acidobacteria-6	o__BPC015	f__,False,0
k__Bacteria	p__Acidobacteria	c__Acidobacteria-6	o__CCU21	f__,False,0
k__Bacteria	p__Acidobacteria	c__Acidobacteria-6	o__iii1-15	__,False,0
k__Bacteria	p__Acidobacteria	c__Acidobacteria-6	o__iii1-15	f__,False,0
k__Bacteria	p__Acidobacteria	c__Acidobacteria-6	o__iii1-15	f__RB40,False,0
k__Bacteria	p__Acidobacteria	c__Acidobacteria-6	o__iii1-15	f__mb2424,False,0
k__Bacteria	p__Acidobacteria	c__Acidobacteriia	o__Acidobacteriales	__,False,0
k__Bacteria	p__Acidobacteria	c__Acidobacteriia	o__Acidobacteriales	f__Koribacteraceae,False,0
k__Bacteria	p__Acidobacteria	c__BPC102	o__	f__,False,0
k__Bacteria	p__Acidobacteria	c__BPC102	o__B110	f__,False,0
k__Bacteria	p__Acidobacteria	c__BPC102	o__MVS-40	f__,False,0
k__Bacteria	p__Acidobacteria	c__DA052	o__E29	f__,False,0
k__Bacteria	p__Acidobacteria	c__DA052	o__Ellin6513	f__,False,0
k__Bacteria	p__Acidobacteria	c__EC1113	o__	f__,False,0
k__Bacteria	p__Acidobacteria	c__PAUC37f	o__	f__,False,0
k__Bacteria	p__Acidobacteria	c__RB25	o__	f__,False,0
k__Bacteria	p__Acidobacteria	c__S035	o__	f__,False,0
k__Bacteria	p__Acidobacteria	c__Solibacteres	o__JH-WHS99	f__,False,0
k__Bacteria	p__Acidobacteria	c__Solibacteres	o__Solibacterales	__,False,0
k__Bacteria	p__Acidobacteria	c__Solibacteres	o__Solibacterales	f__,False,0
k__Bacteria	p__Acidobacteria	c__Solibacteres	o__Solibacterales	f__PAUC26f,False,0
k__Bacteria	p__Acidobacteria	c__Solibacteres	o__Solibacterales	f__Solibacteraceae,False,0
k__Bacteria	p__Acidobacteria	c__Sva0725	o__Sva0725	f__,False,0
k__Bacteria	p__Acidobacteria	c__TM1	o__	f__,False,0
k__Bacteria	p__Acidobacteria	c__[Chloracidobacteria]	__	__,False,0
k__Bacteria	p__Acidobacteria	c__[Chloracidobacteria]	o__11-24	f__,False,0
k__Bacteria	p__Acidobacteria	c__[Chloracidobacteria]	o__	f__,False,0
k__Bacteria	p__Acidobacteria	c__[Chloracidobacteria]	o__PK29	f__,False,0
k__Bacteria	p__Acidobacteria	c__[Chloracidobacteria]	o__RB41	__,False,0
k__Bacteria	p__Acidobacteria	c__[Chloracidobacteria]	o__RB41	f__,False,0
k__Bacteria	p__Acidobacteria	c__[Chloracidobacteria]	o__RB41	f__Ellin6075,False,0
k__Bacteria	p__Acidobacteria	c__iii1-8	o__32-20	f__,False,0
k__Bacteria	p__Acidobacteria	c__iii1-8	o__DS-18	f__,False,0
k__Bacteria	p__Actinobacteria	__	__	__,False,0
k__Bacteria	p__Actinobacteria	c__Acidimicrobiia	o__Acidimicrobiales	__,False,0
k__Bacteria	p__Actinobacteria	c__Acidimicrobiia	o__Acidimicrobiales	f__,False,0
k__Bacteria	p__Actinobacteria	c__Acidimicrobiia	o__Acidimicrobiales	f__AKIW874,False,0
k__Bacteria	p__Actinobacteria	c__Acidimicrobiia	o__Acidimicrobiales	f__C111,False,0
k__Bacteria	p__Actinobacteria	c__Acidimicrobiia	o__Acidimicrobiales	f__EB1017,False,0
k__Bacteria	p__Actinobacteria	c__Acidimicrobiia	o__Acidimicrobiales	f__Iamiaceae,False,0
k__Bacteria	p__Actinobacteria	c__Acidimicrobiia	o__Acidimicrobiales	f__koll13,False,0
k__Bacteria	p__Actinobacteria	c__Actinobacteria	__	__,False,0
k__Bacteria	p__Actinobacteria	c__Actinobacteria	o__Actinomycetales	__,False,0
k__Bacteria	p__Actinobacteria	c__Actinobacteria	o__Actinomycetales	f__,False,0
k__Bacteria	p__Actinobacteria	c__Actinobacteria	o__Actinomycetales	f__Actinosynnemataceae,False,0
k__Bacteria	p__Actinobacteria	c__Actinobacteria	o__Actinomycetales	f__Frankiaceae,False,0
k__Bacteria	p__Actinobacteria	c__Actinobacteria	o__Actinomycetales	f__Glycomycetaceae,False,0
k__Bacteria	p__Actinobacteria	c__Actinobacteria	o__Actinomycetales	f__Microbacteriaceae,False,0
k__Bacteria	p__Actinobacteria	c__Actinobacteria	o__Actinomycetales	f__Micrococcaceae,False,0

k__Bacteria	p__Actinobacteria	c__Actinobacteria	o__Actinomycetales	f__Micromonosporaceae,False,0
k__Bacteria	p__Actinobacteria	c__Actinobacteria	o__Actinomycetales	f__Mycobacteriaceae,False,0
k__Bacteria	p__Actinobacteria	c__Actinobacteria	o__Actinomycetales	f__Nocardiaceae,False,0
k__Bacteria	p__Actinobacteria	c__Actinobacteria	o__Actinomycetales	f__Nocardioidaceae,False,0
k__Bacteria	p__Actinobacteria	c__Actinobacteria	o__Actinomycetales	f__Promicromonosporaceae,False,0
k__Bacteria	p__Actinobacteria	c__Actinobacteria	o__Actinomycetales	f__Pseudonocardiaceae,False,0
k__Bacteria	p__Actinobacteria	c__Actinobacteria	o__Actinomycetales	f__Streptomycetaceae,False,0
k__Bacteria	p__Actinobacteria	c__Actinobacteria	o__Actinomycetales	f__Streptosporangiaceae,False,0
k__Bacteria	p__Actinobacteria	c__Actinobacteria	o__Actinomycetales	f__Thermomonosporaceae,False,0
k__Bacteria	p__Actinobacteria	c__Actinobacteria	o__Micrococcales	f__,False,0
k__Bacteria	p__Actinobacteria	c__MB-A2-108	o__0319-7L14	f__,False,0
k__Bacteria	p__Actinobacteria	c__MB-A2-108	o__	f__,False,0
k__Bacteria	p__Actinobacteria	c__Nitriliruptoria	o__Euzebyales	f__Euzebyaceae,False,0
k__Bacteria	p__Actinobacteria	c__Rubrobacteria	o__Rubrobacterales	f__Rubrobacteraceae,False,0
k__Bacteria	p__Actinobacteria	c__Thermoleophilia	__	__,False,0
k__Bacteria	p__Actinobacteria	c__Thermoleophilia	o__Gaiellales	__,False,0
k__Bacteria	p__Actinobacteria	c__Thermoleophilia	o__Gaiellales	f__,False,0
k__Bacteria	p__Actinobacteria	c__Thermoleophilia	o__Gaiellales	f__AK1AB1_02E,False,0
k__Bacteria	p__Actinobacteria	c__Thermoleophilia	o__Gaiellales	f__Gaiellaceae,False,0
k__Bacteria	p__Actinobacteria	c__Thermoleophilia	o__Solirubrobacterales	__,False,0
k__Bacteria	p__Actinobacteria	c__Thermoleophilia	o__Solirubrobacterales	f__,False,0
k__Bacteria	p__Actinobacteria	c__Thermoleophilia	o__Solirubrobacterales	f__Conexibacteraceae,False,0
k__Bacteria	p__Actinobacteria	c__Thermoleophilia	o__Solirubrobacterales	f__Solirubrobacteraceae,False,0
k__Bacteria	p__Armatimonadetes	c__[Fimbriimonadia]	o__[Fimbriimonadales]	__,False,0
k__Bacteria	p__Armatimonadetes	c__[Fimbriimonadia]	o__[Fimbriimonadales]	f__,False,0
k__Bacteria	p__Armatimonadetes	c__[Fimbriimonadia]	o__[Fimbriimonadales]	f__[Fimbriimonadaceae],False,0
k__Bacteria	p__BRC1	c__PRR-11	o__	f__,False,0
k__Bacteria	p__Bacteroidetes	__	__	__,False,0
k__Bacteria	p__Bacteroidetes	c__Bacteroidia	o__Bacteroidales	f__Bacteroidaceae,False,0
k__Bacteria	p__Bacteroidetes	c__Bacteroidia	o__Bacteroidales	f__Prevotellaceae,False,0
k__Bacteria	p__Bacteroidetes	c__Bacteroidia	o__Bacteroidales	f__[Paraprevotellaceae],False,0
k__Bacteria	p__Bacteroidetes	c__Cytophagia	o__Cytophagales	__,False,0
k__Bacteria	p__Bacteroidetes	c__Cytophagia	o__Cytophagales	f__Cytophagaceae,False,0
k__Bacteria	p__Bacteroidetes	c__Cytophagia	o__Cytophagales	f__[Amoebophilaceae],False,0
k__Bacteria	p__Bacteroidetes	c__Flavobacteriia	o__Flavobacteriales	__,False,0
k__Bacteria	p__Bacteroidetes	c__Flavobacteriia	o__Flavobacteriales	f__Cryomorphaceae,False,0
k__Bacteria	p__Bacteroidetes	c__Flavobacteriia	o__Flavobacteriales	f__[Weeksellaceae],False,0
k__Bacteria	p__Bacteroidetes	c__Sphingobacteriia	o__Sphingobacteriales	__,False,0
k__Bacteria	p__Bacteroidetes	c__Sphingobacteriia	o__Sphingobacteriales	f__,False,0
k__Bacteria	p__Bacteroidetes	c__Sphingobacteriia	o__Sphingobacteriales	f__Sphingobacteriaceae,False,0
k__Bacteria	p__Bacteroidetes	c__VC2_1_Bac22	o__	f__,False,0
k__Bacteria	p__Bacteroidetes	c__[Saprospirae]	o__[Saprospirales]	__,False,0
k__Bacteria	p__Bacteroidetes	c__[Saprospirae]	o__[Saprospirales]	f__,False,0
k__Bacteria	p__Bacteroidetes	c__[Saprospirae]	o__[Saprospirales]	f__Chitinophagaceae,False,0
k__Bacteria	p__Bacteroidetes	c__[Saprospirae]	o__[Saprospirales]	f__Saprospiraceae,False,0
k__Bacteria	p__Chlamydiae	c__Chlamydiia	o__Chlamydiales	__,False,0
k__Bacteria	p__Chlamydiae	c__Chlamydiia	o__Chlamydiales	f__,False,0
k__Bacteria	p__Chlamydiae	c__Chlamydiia	o__Chlamydiales	f__Parachlamydiaceae,False,0
k__Bacteria	p__Chlamydiae	c__Chlamydiia	o__Chlamydiales	f__Rhabdochlamydiaceae,False,0
k__Bacteria	p__Chlorobi	c__	o__	f__,False,0
k__Bacteria	p__Chlorobi	c__BSV26	o__A89	f__,False,0
k__Bacteria	p__Chlorobi	c__BSV26	o__C20	f__,False,0
k__Bacteria	p__Chlorobi	c__BSV26	o__PK329	f__,False,0
k__Bacteria	p__Chlorobi	c__SJA-28	o__	f__,False,0
k__Bacteria	p__Chloroflexi	__	__	__,False,0
k__Bacteria	p__Chloroflexi	c__	o__	f__,False,0
k__Bacteria	p__Chloroflexi	c__Anaerolineae	__	__,False,0
k__Bacteria	p__Chloroflexi	c__Anaerolineae	o__A31	f__,False,0
k__Bacteria	p__Chloroflexi	c__Anaerolineae	o__A31	f__S47,False,0
k__Bacteria	p__Chloroflexi	c__Anaerolineae	o__CFB-26	f__,False,0
k__Bacteria	p__Chloroflexi	c__Anaerolineae	o__Caldilineales	f__Caldilineaceae,False,0
k__Bacteria	p__Chloroflexi	c__Anaerolineae	o__GCA004	f__,False,0
k__Bacteria	p__Chloroflexi	c__Anaerolineae	o__H39	f__,False,0
k__Bacteria	p__Chloroflexi	c__Anaerolineae	o__S0208	f__,False,0
k__Bacteria	p__Chloroflexi	c__Anaerolineae	o__SB-34	f__,False,0
k__Bacteria	p__Chloroflexi	c__Anaerolineae	o__SBR1031	f__A4b,False,0
k__Bacteria	p__Chloroflexi	c__Anaerolineae	o__SBR1031	f__oc28,False,0
k__Bacteria	p__Chloroflexi	c__Anaerolineae	o__envOPS12	f__,False,0
k__Bacteria	p__Chloroflexi	c__Anaerolineae	o__pLW-97	f__,False,0
k__Bacteria	p__Chloroflexi	c__Chloroflexi	o__[Roseiflexales]	__,False,0
k__Bacteria	p__Chloroflexi	c__Chloroflexi	o__[Roseiflexales]	f__[Kouleothrixaceae],False,0

k__Bacteria	p__Chloroflexi	c__Ellin6529	o__	f__,False,0
k__Bacteria	p__Chloroflexi	c__Gitt-GS-136	o__	f__,False,1
k__Bacteria	p__Chloroflexi	c__Ktedonobacteria	__	__,False,0
k__Bacteria	p__Chloroflexi	c__Ktedonobacteria	o__Ktedonobacterales	f__Ktedonobacteraceae,False,0
k__Bacteria	p__Chloroflexi	c__Ktedonobacteria	o__Thermogemmatisporales	f__Thermogemmatisporaceae,False,0
k__Bacteria	p__Chloroflexi	c__P2-11E	o__	f__,False,0
k__Bacteria	p__Chloroflexi	c__S085	o__	f__,False,0
k__Bacteria	p__Chloroflexi	c__SAR202	o__	f__,False,0
k__Bacteria	p__Chloroflexi	c__TK10	__	__,False,0
k__Bacteria	p__Chloroflexi	c__TK10	o__	f__,False,0
k__Bacteria	p__Chloroflexi	c__TK10	o__AKYG885	__,False,0
k__Bacteria	p__Chloroflexi	c__TK10	o__AKYG885	f__,False,0
k__Bacteria	p__Chloroflexi	c__TK10	o__AKYG885	f__5B-12,False,0
k__Bacteria	p__Chloroflexi	c__TK10	o__AKYG885	f__Dolo_23,False,0
k__Bacteria	p__Chloroflexi	c__TK10	o__B07_WMSP1	__,False,0
k__Bacteria	p__Chloroflexi	c__TK10	o__B07_WMSP1	f__,False,0
k__Bacteria	p__Chloroflexi	c__TK17	o__	f__,False,0
k__Bacteria	p__Chloroflexi	c__TK17	o__mle1-48	f__,False,0
k__Bacteria	p__Chloroflexi	c__Thermomicrobia	__	__,False,0
k__Bacteria	p__Chloroflexi	c__Thermomicrobia	o__JG30-KF-CM45	f__,False,0
k__Bacteria	p__Cyanobacteria	c__4C0d-2	o__MLE1-12	f__,False,0
k__Bacteria	p__Cyanobacteria	c__4C0d-2	o__SM1D11	f__,False,0
k__Bacteria	p__Cyanobacteria	c__Chloroplast	o__Stramenopiles	f__,False,0
k__Bacteria	p__Cyanobacteria	c__Chloroplast	o__Streptophyta	f__,False,0
k__Bacteria	p__Cyanobacteria	c__ML635J-21	o__	f__,False,0
k__Bacteria	p__Elusimicrobia	__	__	__,False,0
k__Bacteria	p__Elusimicrobia	c__Elusimicrobia	__	__,False,0
k__Bacteria	p__Elusimicrobia	c__Elusimicrobia	o__Elusimicrobiales	f__,False,0
k__Bacteria	p__Elusimicrobia	c__Elusimicrobia	o__FAC88	f__,False,0
k__Bacteria	p__Elusimicrobia	c__Elusimicrobia	o__IIb	f__,False,0
k__Bacteria	p__Elusimicrobia	c__Elusimicrobia	o__MVP-88	f__,False,0
k__Bacteria	p__Elusimicrobia	c__Endomicrobia	o__	f__,False,0
k__Bacteria	p__Fibrobacteres	c__Fibrobacteria	o__258ds10	f__,False,0
k__Bacteria	p__Firmicutes	__	__	__,False,0
k__Bacteria	p__Firmicutes	c__Bacilli	o__Bacillales	__,False,0
k__Bacteria	p__Firmicutes	c__Bacilli	o__Bacillales	f__Bacillaceae,False,0
k__Bacteria	p__Firmicutes	c__Bacilli	o__Bacillales	f__Paenibacillaceae,False,0
k__Bacteria	p__Firmicutes	c__Bacilli	o__Bacillales	f__Planococcaceae,False,0
k__Bacteria	p__Firmicutes	c__Bacilli	o__Bacillales	f__Staphylococcaceae,False,0
k__Bacteria	p__Firmicutes	c__Bacilli	o__Bacillales	f__Thermoactinomycetaceae,False,0
k__Bacteria	p__Firmicutes	c__Bacilli	o__Lactobacillales	f__Aerococcaceae,False,0
k__Bacteria	p__Firmicutes	c__Bacilli	o__Lactobacillales	f__Carnobacteriaceae,False,0
k__Bacteria	p__Firmicutes	c__Bacilli	o__Lactobacillales	f__Streptococcaceae,False,0
k__Bacteria	p__Firmicutes	c__Clostridia	__	__,False,0
k__Bacteria	p__Firmicutes	c__Clostridia	o__Clostridiales	__,False,0
k__Bacteria	p__Firmicutes	c__Clostridia	o__Clostridiales	f__Clostridiaceae,False,0
k__Bacteria	p__Firmicutes	c__Clostridia	o__Clostridiales	f__Gracilibacteraceae,False,0
k__Bacteria	p__Firmicutes	c__Clostridia	o__Clostridiales	f__Lachnospiraceae,False,0
k__Bacteria	p__Firmicutes	c__Clostridia	o__Clostridiales	f__Peptococcaceae,False,0
k__Bacteria	p__Firmicutes	c__Clostridia	o__Clostridiales	f__Ruminococcaceae,False,0
k__Bacteria	p__Firmicutes	c__Clostridia	o__Clostridiales	f__Symbiobacteriaceae,False,0
k__Bacteria	p__Firmicutes	c__Clostridia	o__Clostridiales	f__Veillonellaceae,False,0
k__Bacteria	p__Firmicutes	c__Clostridia	o__Clostridiales	f__[Mogibacteriaceae],False,0
k__Bacteria	p__Firmicutes	c__Clostridia	o__Clostridiales	f__[Tissierellaceae],False,0
k__Bacteria	p__Firmicutes	c__Clostridia	o__OPB54	f__,False,0
k__Bacteria	p__GAL15	c__	o__	f__,False,0
k__Bacteria	p__GN02	c__GKS2-174	o__	f__,False,0
k__Bacteria	p__GN04	c__	o__	f__,False,0
k__Bacteria	p__GN04	c__GN15	o__	f__,False,0
k__Bacteria	p__GN04	c__MSB-5A5	o__	f__,False,0
k__Bacteria	p__Gemmatimonadetes	__	__	__,False,0
k__Bacteria	p__Gemmatimonadetes	c__	o__	f__,False,0
k__Bacteria	p__Gemmatimonadetes	c__Gemm-1	o__	f__,False,0
k__Bacteria	p__Gemmatimonadetes	c__Gemm-2	o__	f__,False,0
k__Bacteria	p__Gemmatimonadetes	c__Gemm-5	o__	f__,False,0
k__Bacteria	p__Gemmatimonadetes	c__Gemmatimonadetes	__	__,False,0
k__Bacteria	p__Gemmatimonadetes	c__Gemmatimonadetes	o__	f__,False,0
k__Bacteria	p__Gemmatimonadetes	c__Gemmatimonadetes	o__C114	f__,False,0
k__Bacteria	p__Gemmatimonadetes	c__Gemmatimonadetes	o__KD8-87	f__,False,0
k__Bacteria	p__MVP-21	c__	o__	f__,False,0
k__Bacteria	p__NC10	c__12-24	o__MIZ17	f__,False,0

k__Bacteria	p__NKB19	c__	o__	f__,False,0
k__Bacteria	p__NKB19	c__TSBW08	o__	f__,False,0
k__Bacteria	p__Nitrospirae	c__Nitrospira	o__Nitrospirales	__,False,0
k__Bacteria	p__Nitrospirae	c__Nitrospira	o__Nitrospirales	f__,False,0
k__Bacteria	p__Nitrospirae	c__Nitrospira	o__Nitrospirales	f__0319-6A21,False,0
k__Bacteria	p__Nitrospirae	c__Nitrospira	o__Nitrospirales	f__Nitrospiraceae,False,0
k__Bacteria	p__Nitrospirae	c__Nitrospira	o__Nitrospirales	f__[Leptospirillaceae],False,0
k__Bacteria	p__OD1	__	__	__,False,0
k__Bacteria	p__OD1	c__ABY1	o__	f__,False,0
k__Bacteria	p__OD1	c__Mb-NB09	o__	f__,False,0
k__Bacteria	p__OD1	c__SM2F11	o__	f__,False,0
k__Bacteria	p__OD1	c__ZB2	o__	f__,False,0
k__Bacteria	p__OP11	__	__	__,False,0
k__Bacteria	p__OP11	c__WCHB1-64	o__d153	f__,False,0
k__Bacteria	p__OP3	c__PBS-25	o__	f__,False,0
k__Bacteria	p__OP3	c__koll11	o__	f__,False,0
k__Bacteria	p__OP3	c__koll11	o__GIF10	f__kpj58rc,False,0
k__Bacteria	p__Planctomycetes	__	__	__,False,0
k__Bacteria	p__Planctomycetes	c__	o__	f__,False,0
k__Bacteria	p__Planctomycetes	c__BD7-11	o__	f__,False,0
k__Bacteria	p__Planctomycetes	c__OM190	o__CL500-15	f__,False,0
k__Bacteria	p__Planctomycetes	c__OM190	o__agg27	f__,False,0
k__Bacteria	p__Planctomycetes	c__Phycisphaerae	o__CCM11a	f__,False,0
k__Bacteria	p__Planctomycetes	c__Phycisphaerae	o__Phycisphaerales	__,False,0
k__Bacteria	p__Planctomycetes	c__Phycisphaerae	o__Phycisphaerales	f__,False,0
k__Bacteria	p__Planctomycetes	c__Phycisphaerae	o__Pla1	f__,False,0
k__Bacteria	p__Planctomycetes	c__Phycisphaerae	o__S-70	f__,False,0
k__Bacteria	p__Planctomycetes	c__Phycisphaerae	o__WD2101	f__,False,0
k__Bacteria	p__Planctomycetes	c__Pla3	o__	f__,False,0
k__Bacteria	p__Planctomycetes	c__Pla4	o__	f__,False,0
k__Bacteria	p__Planctomycetes	c__Planctomycetia	__	__,False,0
k__Bacteria	p__Planctomycetes	c__Planctomycetia	o__B97	f__,False,0
k__Bacteria	p__Planctomycetes	c__Planctomycetia	o__Gemmatales	__,False,0
k__Bacteria	p__Planctomycetes	c__Planctomycetia	o__Gemmatales	f__Gemmataceae,False,0
k__Bacteria	p__Planctomycetes	c__Planctomycetia	o__Gemmatales	f__Isosphaeraceae,False,0
k__Bacteria	p__Planctomycetes	c__Planctomycetia	o__Pirellulales	f__Pirellulaceae,False,1
k__Bacteria	p__Planctomycetes	c__Planctomycetia	o__Planctomycetales	f__Planctomycetaceae,False,0
k__Bacteria	p__Proteobacteria	__	__	__,False,0
k__Bacteria	p__Proteobacteria	c__	o__	f__,False,0
k__Bacteria	p__Proteobacteria	c__Alphaproteobacteria	__	__,False,0
k__Bacteria	p__Proteobacteria	c__Alphaproteobacteria	o__BD7-3	f__,False,0
k__Bacteria	p__Proteobacteria	c__Alphaproteobacteria	o__Caulobacterales	f__Caulobacteraceae,False,0
k__Bacteria	p__Proteobacteria	c__Alphaproteobacteria	o__Ellin329	f__,False,0
k__Bacteria	p__Proteobacteria	c__Alphaproteobacteria	o__Rhizobiales	__,False,0
k__Bacteria	p__Proteobacteria	c__Alphaproteobacteria	o__Rhizobiales	f__,False,0
k__Bacteria	p__Proteobacteria	c__Alphaproteobacteria	o__Rhizobiales	f__Bradyrhizobiaceae,False,0
k__Bacteria	p__Proteobacteria	c__Alphaproteobacteria	o__Rhizobiales	f__Brucellaceae,False,0
k__Bacteria	p__Proteobacteria	c__Alphaproteobacteria	o__Rhizobiales	f__Hyphomicrobiaceae,False,0
k__Bacteria	p__Proteobacteria	c__Alphaproteobacteria	o__Rhizobiales	f__Methylocystaceae,False,0
k__Bacteria	p__Proteobacteria	c__Alphaproteobacteria	o__Rhizobiales	f__Phyllobacteriaceae,False,0
k__Bacteria	p__Proteobacteria	c__Alphaproteobacteria	o__Rhizobiales	f__Rhizobiaceae,False,0
k__Bacteria	p__Proteobacteria	c__Alphaproteobacteria	o__Rhizobiales	f__Rhodobiaceae,False,0
k__Bacteria	p__Proteobacteria	c__Alphaproteobacteria	o__Rhizobiales	f__Xanthobacteraceae,False,0
k__Bacteria	p__Proteobacteria	c__Alphaproteobacteria	o__Rhodobacterales	f__Hyphomonadaceae,False,0
k__Bacteria	p__Proteobacteria	c__Alphaproteobacteria	o__Rhodobacterales	f__Rhodobacteraceae,False,0
k__Bacteria	p__Proteobacteria	c__Alphaproteobacteria	o__Rhodospirillales	__,False,0
k__Bacteria	p__Proteobacteria	c__Alphaproteobacteria	o__Rhodospirillales	f__,False,0
k__Bacteria	p__Proteobacteria	c__Alphaproteobacteria	o__Rhodospirillales	f__Acetobacteraceae,False,0
k__Bacteria	p__Proteobacteria	c__Alphaproteobacteria	o__Rhodospirillales	f__Rhodospirillaceae,False,0
k__Bacteria	p__Proteobacteria	c__Alphaproteobacteria	o__Rickettsiales	f__,False,0
k__Bacteria	p__Proteobacteria	c__Alphaproteobacteria	o__Rickettsiales	f__Holosporaceae,False,0
k__Bacteria	p__Proteobacteria	c__Alphaproteobacteria	o__Rickettsiales	f__Rickettsiaceae,False,0
k__Bacteria	p__Proteobacteria	c__Alphaproteobacteria	o__Rickettsiales	f__mitochondria,False,0
k__Bacteria	p__Proteobacteria	c__Alphaproteobacteria	o__Sphingomonadales	__,False,0
k__Bacteria	p__Proteobacteria	c__Alphaproteobacteria	o__Sphingomonadales	f__Erythrobacteraceae,False,0
k__Bacteria	p__Proteobacteria	c__Alphaproteobacteria	o__Sphingomonadales	f__Sphingomonadaceae,False,0
k__Bacteria	p__Proteobacteria	c__Betaproteobacteria	__	__,False,0
k__Bacteria	p__Proteobacteria	c__Betaproteobacteria	o__	f__,False,0
k__Bacteria	p__Proteobacteria	c__Betaproteobacteria	o__A21b	f__EB1003,False,0
k__Bacteria	p__Proteobacteria	c__Betaproteobacteria	o__Burkholderiales	__,False,0
k__Bacteria	p__Proteobacteria	c__Betaproteobacteria	o__Burkholderiales	f__,False,0

k__Bacteria	p__Proteobacteria	c__Betaproteobacteria	o__Burkholderiales	f__Alcaligenaceae,False,0
k__Bacteria	p__Proteobacteria	c__Betaproteobacteria	o__Burkholderiales	f__Burkholderiaceae,False,0
k__Bacteria	p__Proteobacteria	c__Betaproteobacteria	o__Burkholderiales	f__Comamonadaceae,False,0
k__Bacteria	p__Proteobacteria	c__Betaproteobacteria	o__Burkholderiales	f__Oxalobacteraceae,False,0
k__Bacteria	p__Proteobacteria	c__Betaproteobacteria	o__Ellin6067	f__,False,0
k__Bacteria	p__Proteobacteria	c__Betaproteobacteria	o__IS-44	f__,False,0
k__Bacteria	p__Proteobacteria	c__Betaproteobacteria	o__MND1	f__,False,0
k__Bacteria	p__Proteobacteria	c__Betaproteobacteria	o__Methylophilales	f__Methylophilaceae,False,0
k__Bacteria	p__Proteobacteria	c__Betaproteobacteria	o__Neisseriales	f__Neisseriaceae,False,0
k__Bacteria	p__Proteobacteria	c__Betaproteobacteria	o__Procabacteriales	f__Procabacteriaceae,False,0
k__Bacteria	p__Proteobacteria	c__Betaproteobacteria	o__SC-I-84	f__,False,0
k__Bacteria	p__Proteobacteria	c__Deltaproteobacteria	__	__,False,0
k__Bacteria	p__Proteobacteria	c__Deltaproteobacteria	o__Bdellovibrionales	f__Bacteriovoracaceae,False,0
k__Bacteria	p__Proteobacteria	c__Deltaproteobacteria	o__Bdellovibrionales	f__Bdellovibrionaceae,False,0
k__Bacteria	p__Proteobacteria	c__Deltaproteobacteria	o__Desulfuromonadales	f__Geobacteraceae,False,0
k__Bacteria	p__Proteobacteria	c__Deltaproteobacteria	o__FAC87	f__,False,0
k__Bacteria	p__Proteobacteria	c__Deltaproteobacteria	o__GMD14H09	f__,False,0
k__Bacteria	p__Proteobacteria	c__Deltaproteobacteria	o__MIZ46	f__,False,0
k__Bacteria	p__Proteobacteria	c__Deltaproteobacteria	o__Myxococcales	__,False,0
k__Bacteria	p__Proteobacteria	c__Deltaproteobacteria	o__Myxococcales	f__,False,0
k__Bacteria	p__Proteobacteria	c__Deltaproteobacteria	o__Myxococcales	f__0319-6G20,False,0
k__Bacteria	p__Proteobacteria	c__Deltaproteobacteria	o__Myxococcales	f__Cystobacteraceae,False,0
k__Bacteria	p__Proteobacteria	c__Deltaproteobacteria	o__Myxococcales	f__Cystobacterineae,False,0
k__Bacteria	p__Proteobacteria	c__Deltaproteobacteria	o__Myxococcales	f__Haliangiaceae,False,0
k__Bacteria	p__Proteobacteria	c__Deltaproteobacteria	o__Myxococcales	f__Myxococcaceae,False,0
k__Bacteria	p__Proteobacteria	c__Deltaproteobacteria	o__Myxococcales	f__Nannocystaceae,False,0
k__Bacteria	p__Proteobacteria	c__Deltaproteobacteria	o__Myxococcales	f__OM27,False,0
k__Bacteria	p__Proteobacteria	c__Deltaproteobacteria	o__Myxococcales	f__Polyangiaceae,False,0
k__Bacteria	p__Proteobacteria	c__Deltaproteobacteria	o__NB1-j	__,False,0
k__Bacteria	p__Proteobacteria	c__Deltaproteobacteria	o__NB1-j	f__,False,0
k__Bacteria	p__Proteobacteria	c__Deltaproteobacteria	o__NB1-j	f__JTB38,False,0
k__Bacteria	p__Proteobacteria	c__Deltaproteobacteria	o__NB1-j	f__MND4,False,0
k__Bacteria	p__Proteobacteria	c__Deltaproteobacteria	o__NB1-j	f__NB1-i,False,0
k__Bacteria	p__Proteobacteria	c__Deltaproteobacteria	o__Spirobacillales	f__,False,0
k__Bacteria	p__Proteobacteria	c__Deltaproteobacteria	o__Sva0853	f__JTB36,False,0
k__Bacteria	p__Proteobacteria	c__Deltaproteobacteria	o__Syntrophobacterales	__,False,0
k__Bacteria	p__Proteobacteria	c__Deltaproteobacteria	o__Syntrophobacterales	f__Syntrophobacteraceae,False,0
k__Bacteria	p__Proteobacteria	c__Deltaproteobacteria	o__[Entotheonellales]	f__[Entotheonellaceae],False,0
k__Bacteria	p__Proteobacteria	c__Gammaproteobacteria	__	__,False,0
k__Bacteria	p__Proteobacteria	c__Gammaproteobacteria	o__Alteromonadales	f__125ds10,False,0
k__Bacteria	p__Proteobacteria	c__Gammaproteobacteria	o__Alteromonadales	f__211ds20,False,0
k__Bacteria	p__Proteobacteria	c__Gammaproteobacteria	o__Chromatiales	__,False,0
k__Bacteria	p__Proteobacteria	c__Gammaproteobacteria	o__Enterobacteriales	f__Enterobacteriaceae,False,0
k__Bacteria	p__Proteobacteria	c__Gammaproteobacteria	o__HTCC2188	f__HTCC2089,False,0
k__Bacteria	p__Proteobacteria	c__Gammaproteobacteria	o__Legionellales	__,False,0
k__Bacteria	p__Proteobacteria	c__Gammaproteobacteria	o__Legionellales	f__,False,0
k__Bacteria	p__Proteobacteria	c__Gammaproteobacteria	o__Legionellales	f__Coxiellaceae,False,0
k__Bacteria	p__Proteobacteria	c__Gammaproteobacteria	o__Legionellales	f__Legionellaceae,False,0
k__Bacteria	p__Proteobacteria	c__Gammaproteobacteria	o__Oceanospirillales	f__Halomonadaceae,False,0
k__Bacteria	p__Proteobacteria	c__Gammaproteobacteria	o__Pasteurellales	f__Pasteurellaceae,False,0
k__Bacteria	p__Proteobacteria	c__Gammaproteobacteria	o__Pseudomonadales	f__Moraxellaceae,False,0
k__Bacteria	p__Proteobacteria	c__Gammaproteobacteria	o__Pseudomonadales	f__Pseudomonadaceae,False,0
k__Bacteria	p__Proteobacteria	c__Gammaproteobacteria	o__Thiotrichales	f__Piscirickettsiaceae,False,0
k__Bacteria	p__Proteobacteria	c__Gammaproteobacteria	o__Vibrionales	f__Vibrionaceae,False,0
k__Bacteria	p__Proteobacteria	c__Gammaproteobacteria	o__Xanthomonadales	f__Sinobacteraceae,False,0
k__Bacteria	p__Proteobacteria	c__Gammaproteobacteria	o__Xanthomonadales	f__Xanthomonadaceae,False,0
k__Bacteria	p__Proteobacteria	c__TA18	o__PHOS-HD29	f__,False,0
k__Bacteria	p__SBR1093	c__	o__	f__,False,0
k__Bacteria	p__Spirochaetes	c__[Leptospirae]	o__[Leptospirales]	f__Leptospiraceae,False,0
k__Bacteria	p__TM6	__	__	__,False,0
k__Bacteria	p__TM6	c__SBRH58	o__	f__,False,0
k__Bacteria	p__TM6	c__SJA-4	__	__,False,0
k__Bacteria	p__TM6	c__SJA-4	o__	f__,False,0
k__Bacteria	p__TM6	c__SJA-4	o__S1198	f__,False,0
k__Bacteria	p__TM6	c__SJA-4	o__YJF2-48	f__,False,0
k__Bacteria	p__TM7	__	__	__,False,0
k__Bacteria	p__TM7	c__	o__	f__,False,0
k__Bacteria	p__TM7	c__MJK10	o__	f__,False,0
k__Bacteria	p__TM7	c__SC3	o__	f__,False,0
k__Bacteria	p__TM7	c__TM7-1	o__	f__,False,0
k__Bacteria	p__TM7	c__TM7-3	o__	f__,False,0

k__Bacteria	p__TM7	c__TM7-3	o__EW055	f__,False,0
k__Bacteria	p__TM7	c__TM7-3	o__I025	f__,False,0
k__Bacteria	p__Tenericutes	c__Mollicutes	o__Anaeroplasmatales	f__Anaeroplasmataceae,False,0
k__Bacteria	p__Verrucomicrobia	__	__	__,False,0
k__Bacteria	p__Verrucomicrobia	c__Opitutae	__	__,False,0
k__Bacteria	p__Verrucomicrobia	c__Opitutae	o__HA64	f__,False,0
k__Bacteria	p__Verrucomicrobia	c__Opitutae	o__Opitutales	f__Opitutaceae,False,0
k__Bacteria	p__Verrucomicrobia	c__Verrucomicrobiae	o__Verrucomicrobiales	f__Verrucomicrobiaceae,False,0
k__Bacteria	p__Verrucomicrobia	c__[Methylacidiphilae]	o__Methylacidiphilales	f__LD19,False,0
k__Bacteria	p__Verrucomicrobia	c__[Methylacidiphilae]	o__S-BQ2-57	f__,False,0
k__Bacteria	p__Verrucomicrobia	c__[Pedosphaerae]	o__[Pedosphaerales]	__,False,0
k__Bacteria	p__Verrucomicrobia	c__[Pedosphaerae]	o__[Pedosphaerales]	f__,False,0
k__Bacteria	p__Verrucomicrobia	c__[Pedosphaerae]	o__[Pedosphaerales]	f__Ellin515,False,0
k__Bacteria	p__Verrucomicrobia	c__[Pedosphaerae]	o__[Pedosphaerales]	f__Ellin517,False,0
k__Bacteria	p__Verrucomicrobia	c__[Pedosphaerae]	o__[Pedosphaerales]	f__OPB35,False,0
k__Bacteria	p__Verrucomicrobia	c__[Pedosphaerae]	o__[Pedosphaerales]	f__auto67_4W,False,0
k__Bacteria	p__Verrucomicrobia	c__[Spartobacteria]	o__[Chthoniobacterales]	f__[Chthoniobacteraceae],False,0
k__Bacteria	p__WS2	c__SHA-109	o__	f__,False,0
k__Bacteria	p__WS3	c__PRR-12	o__LD1-PA13	f__,False,0
k__Bacteria	p__WS3	c__PRR-12	o__Sediment-1	f__,False,0
k__Bacteria	p__WS3	c__PRR-12	o__Sediment-1	f__PRR-10,False,0
k__Bacteria	p__WS3	c__PRR-12	o__wb1_H11	f__,False,0
k__Bacteria	p__WS6	c__B142	o__	f__,False,0
k__Bacteria	p__[Caldithrix]	c__KSB1	o__Ucn15732	f__,False,0
