# Supplementary material for: Quantitative and qualitative evaluation of the impact of the G2 enhancer, bead sizes and lysing tubes on the bacterial community composition during DNA extraction from recalcitrant soil core samples based on community sequencing and qPCR
Source: PLoS One. 2019 Apr 11;14(4):e0200979. doi: 10.1371/journal.pone.0200979 (PMC6459482; doi:10.1371/journal.pone.0200979)
Supplement: S1 Fig — (PDF) [file pone.0200979.s009.pdf]

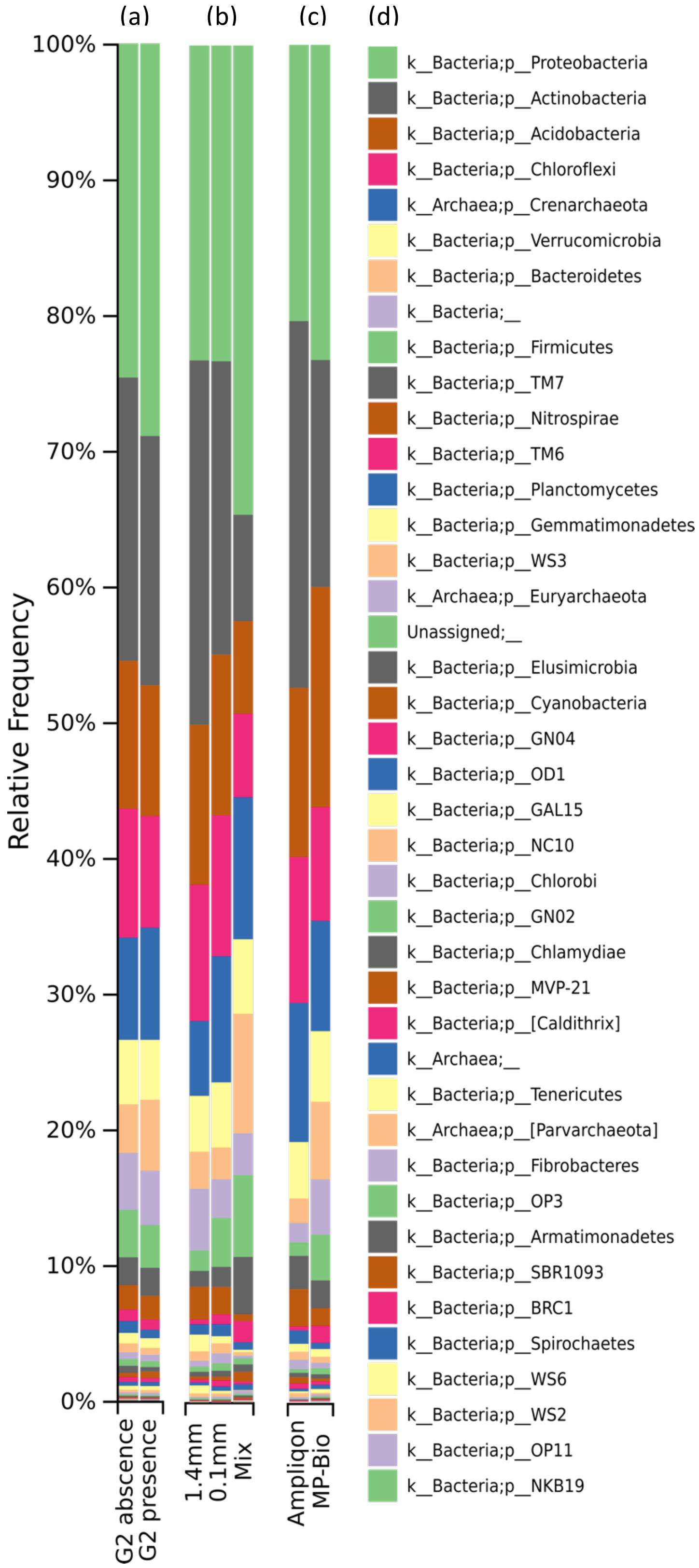

S1 Fig. Compositional bar charts on phylum resolution. (a) G2 presence/absence, (b) beads-effect, (c) tube suppliers and (d) taxonomy. The chromatic order in the label starts from the top-most abundant and re-cycle every eight taxa.
